# Duration of untreated psychosis and involuntary hospitalization in first-episode psychosis

**DOI:** 10.1192/j.eurpsy.2024.1521

**Published:** 2024-08-27

**Authors:** H. J. Gomes, R. A. Moreira, J. P. Correia, E. Maldonado, J. M. Barros, J. R. Gomes

**Affiliations:** Unidade Local de Saúde do Nordeste, Bragança, Portugal

## Abstract

**Introduction:**

Duration of untreated psychosis (DUP) is defined as the time between the onset of psychotic symptoms and the initiation of appropriate treatment. DUP has been the subject of intensive research to understand how it is associated with a poorer prognosis in patients with first-episode psychosis (FEP). Involuntary treatment is often necessary in the context of FEP.

**Objectives:**

To characterize the relationship between the duration of untreated psychosis (DUP) and the type of hospitalization (voluntary versus involuntary) in patients admitted for FEP.

**Methods:**

We conducted a retrospective observational study, collecting data from patients admitted between January 2019 and December 2022, in the psychiatric unit at our hospital in Bragança, Portugal. We used the information recorded in the clinical records and statistical analysis of the data was performed using the SPSS program.

**Results:**

Over the 4-year study period, 81 patients with first-episode psychotic symptoms at admission were selected. The average age was 46.98 years, with a slight male predominance. 46.9% (n=38) were admitted involuntarily, and 53.1% (n=43) were admitted voluntarily. The average DUP was 73 days. DUP was 95.92 days for patients admitted involuntarily and 54.72 days for voluntary admission. This difference was not statistically significant.

**Conclusions:**

There was a longer DUP in patients admitted involuntarily, although this association was not statistically significant. However, it is important to emphasize that involuntary hospitalization is frequently linked to more severe cases and poorer prognosis. Therefore, recognizing psychotic symptoms as early as possible is essential to facilitate prompt identification and effective treatment for patients experiencing their first episode of psychosis, ultimately leading to an improved prognosis.

**Disclosure of Interest:**

None Declared

